# Moderately decreasing fertilizer in fields does not reduce populations of cereal aphids but maximizes fitness of parasitoids

**DOI:** 10.1038/s41598-021-81855-8

**Published:** 2021-01-28

**Authors:** Fei Qiao, Quan-Feng Yang, Rui-Xing Hou, Ke-Ning Zhang, Jing Li, Feng Ge, Fang Ouyang

**Affiliations:** 1 State Key Laboratory of Integrated Management of Pest and Rodents, Institute of Zoology, Chinese Academy of Sciences, Beijing, 100101 China; 2 Key Laboratory of Ecosystem Network Observation and Modeling, Institute of Geographic Sciences and Natural Resources Research, Chinese Academy of Sciences, Beijing, 100101 China; 3 CAS Center for Excellence in Biotic Interactions, University of Chinese Academy of Sciences, Beijing, 100049 China

**Keywords:** Agroecology, Entomology

## Abstract

Examination of the tradeoff between the extent of decreasing nitrogen input and pest suppression is crucial for maintaining the balance between essential yield and an efficient, sustainable pest control strategy. In this study, an experiment with four manipulated nitrogen fertilizer levels (70, 140, 210, and 280 kg N ha^−1^ = conventional level) was conducted to explore the effects of decreasing nitrogen on cereal aphids (*Sitobion avenae* and *Rhopalosiphum padi*) (Hemiptera: Aphididae), Aphidiinae parasitoids (Hymenoptera: Braconidae: Aphidiinae), and body sizes of parasitoids. The results indicated that nitrogen application, in the range of 70–280 kg N ha^−1^, has the potential to impact the populations of cereal aphids and their parasitoids. However, both differences between densities of cereal aphids and their parasitoids in moderate (140–210 kg N ha^−1^) and those in high nitrogen input (280 kg N ha^−1^) were not significant, and the parasitism rate was also unaffected. A higher parasitism rate reduced population growth of the cereal aphid (*S. avenae*). Additionally, a moderate decrease of nitrogen fertilizer from 280 to 140–210 kg N ha^−1^ maximized the body sizes of Aphidiinae parasitoids, indicating that a moderate decrease of nitrogen fertilizer could facilitate biocontrol of cereal aphid by parasitoids in the near future. We conclude that a moderate decrease in nitrogen application, from 280 to 140–210 kg N ha^−1^, does not quantitatively impact the densities of cereal aphids or the parasitism rate but can qualitatively maximize the fitness of the parasitoids.

## Introduction

Agricultural intensification driven predominantly by human activity is beneficial to increase crop yields to meet human needs for food. Nitrogen fertilizer as a critical ingredient of agricultural intensification plays a crucial role in agricultural production^1,2^. However, overuse of nitrogen fertilizer has detrimental impacts on the global environment, including induction of pest outbreaks, loss of biodiversity and degradation of biological controls^3–5^. Appropriate nitrogen fertilizer is important to implement sustainable agricultural development. Optimizing application of nitrogen fertilizer to maintain agricultural production and lower the pest impact is of global concern.

Winter wheat is the most essential grain crop globally. At present, the magnitude of nitrogen input in wheat field usually features about 200 kg N ha^−1^ in Europe^2,6^, and 300 kg N ha^−1^ in the North China Plain, the single most important wheat production region in China^7,8^. A recent study considered the balance between wheat yield and environmental quality, the later featured leaching of soil nitrate into the deeper soil layers, recommending 120–170 kg N ha^−1^ as an optimal application level^2,9^. Accordingly, the range of 120–170 kg N ha^−1^ could be employed as a preliminary criterion to sustain acceptable grain yield while reducing detrimental environment concerns. It is necessary to evaluate the effect of such a moderate amount of nitrogen input on the population of the main insect pests and their natural enemies in winter wheat.

Cereal aphids, including *Sitobion avenae* and *Rhopalosiphum padi* (Hemiptera: Aphididae), cause serious economic yield losses of winter wheat. In terms of cultural control, the effects of nitrogen application on the populations of cereal aphids present mixed results^5,10–12^. For example, both population of cereal aphids (*S. avenae* and *R. padi*) are positively responsive to nitrogen levels in the range of 115–170 kg N ha^−1^ in northern China^5^. In contrast, nitrogen fertilizer has a negative influence on the density of cereal aphid (*S. avenae*) in Germany^11^. Considering that the levels of nitrogen input in different research studies are varied, it is reasonable to assume that adequate coverage of nitrogen levels accounts for the observed variation. Therefore, it is urgently necessary to conduct related research that considers greater coverage of nitrogen levels to ascertain to what extent, if any, decreasing nitrogen fertilizer could markedly reduce the populations of cereal aphids.

In terms of biological control, Aphidiinae parasitoids play crucial roles in natural aphid control^5,13,14^. Two Aphidiinae parasitoid species, *Aphidius uzbekistanicus* and *A. gifuensis* (Hymenoptera: Braconidae: Aphidiinae), predominate in wheat growing area of northern China^15^. These Aphidiinae parasitoids share both aphid host and hyperparasitoid species^5,15^, and are identified as controphic species^16^. The effects of nitrogen fertilizer on biocontrol by Aphidiinae parasitoids on cereal aphids are varied^5,10,17^. For example, the parasitism rate in plots exposed to fertilizer is lower compared with that in fertilizer-free plots in Germany^11^, while the parasitism rate increased with nitrogen input in northern China^5^. A manipulation study in the laboratory showed that parasitism rate remained unaffected by nitrogen level^18^. Due to the phenology of cereal aphids and their Aphidiinae parasitoids being closely associated with investigated regions, combined with the fact that adequate coverage of nitrogen levels plays an important role in biocontrol, it is unknown how nitrogen level affects the populations cereal aphids and Aphidiinae parasitoids in cereal fields in northern China.

The body size of parasitoids, especially solitary koinobiont parasitoids, impacts the interaction strength and the structure of the host-parasitoid network^19,20^. The hind tibia length and the head width of parasitoids are positively associated with fecundity and fitness of parasitoid adults as well as their offspring^20^. Thus, the body sizes of parasitoids characterize the fitness of parasitoids and have been adopted to evaluate the fitness of parasitoids^19–21^. It has been verified that the body sizes of parasitoids are stimulated by nitrogen fertilizer application at low aphid densities in the laboratory^22,23^. However, it is unknown whether such effects of nitrogen fertilizer on the body sizes of parasitoids occur in wheat fields.

Systematic tests were conducted to explore the effects of decreasing nitrogen on cereal aphids and Aphidiinae parasitoids in this study. This study combined field investigation in northern China with measurement of body sizes of parasitoids, under four manipulated nitrogen fertilizer levels (70, 140, 210, and 280 kg N ha^−1^). Considering the precondition of ensuring the essential grain yield, the goals of this study were to determine to what extent, if any, decreasing nitrogen fertilizer could decrease the densities of cereal aphids and to examine the incidental effects of decreasing nitrogen fertilizer on quantitative and qualitative aspects of the parasitoids. The specific goals include ascertaining (1) the effect of decreasing nitrogen fertilizer on the densities of cereal aphids; (2) the effect of decreasing nitrogen fertilizer on the density of parasitoids and the parasitism rate; (3) the effect of decreasing nitrogen fertilizer on the body sizes of parasitoids; (4) the effect of parasitism rate on the population growth of cereal aphid.

## Results

### Effect of decreasing nitrogen fertilizer on the densities of cereal aphids

**Figure 1:**
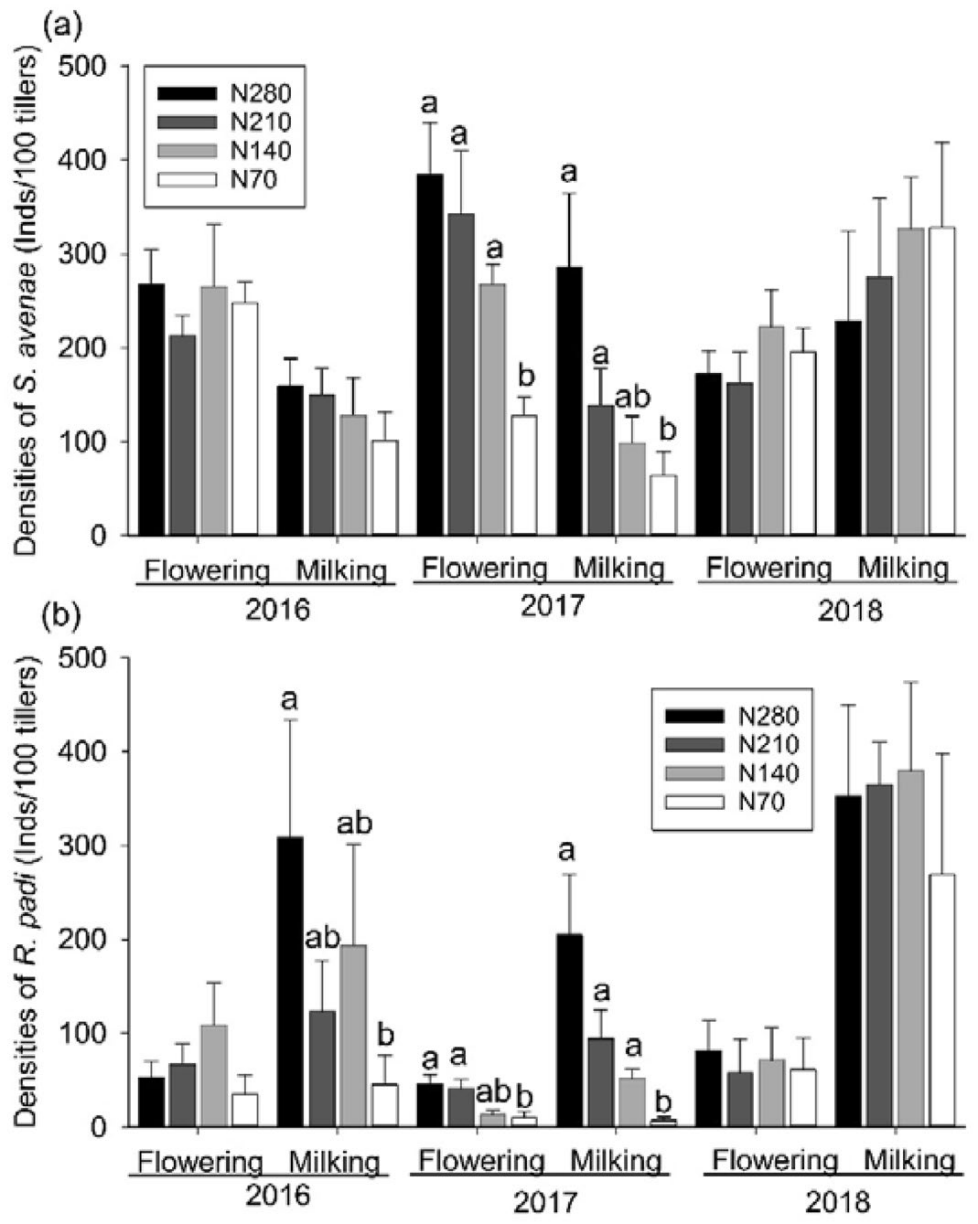
Densities of cereal aphids (**a**: *S. avenae*; **b**: *R. padi*) (means + SE) in nitrogen fertilizer plots at the flowering and milking phases during 2016–2018.

The densities of cereal aphid *S. avenae* were decreased significantly by decreasing nitrogen fertilizer at both flowering (*F*_3, 22_ = 8.62, *P* = 0.001) and milking (*F*_3, 22_ = 4.49, *P* = 0.013) phases in 2017. However, there were no significant differences between *S. avenae* densities in the N280 plots and those in the N210 or N140 plots (Fig. 1a). The differences of *S. avenae* densities among all nitrogen levels were not significant in either 2016 or 2018, at either flowering or milking phases (Fig. 1a).

The densities of the cereal aphid *R. padi* in the N70 plots were significantly lower than those in the N280 plots at the milking phase in 2016 (*F*_3, 22_ = 3.61, *P* = 0.029), as well as at the flowering (*F*_3, 22_ = 3.64, *P* = 0.028) and milking phases (*F*_3, 22_ = 10.98, *P* < 0.001) in 2017. However, the differences between *R. padi* densities in the N280 plots and those in the N210 or N140 plots were nonsignificant at any phase, including in 2018 when nitrogen levels did not have obvious effects on the densities of *R. padi* at any phase (Fig. 1b).

### Effect of decreasing nitrogen fertilizer on the densities of parasitoids and the parasitism rate

**Figure 2:**
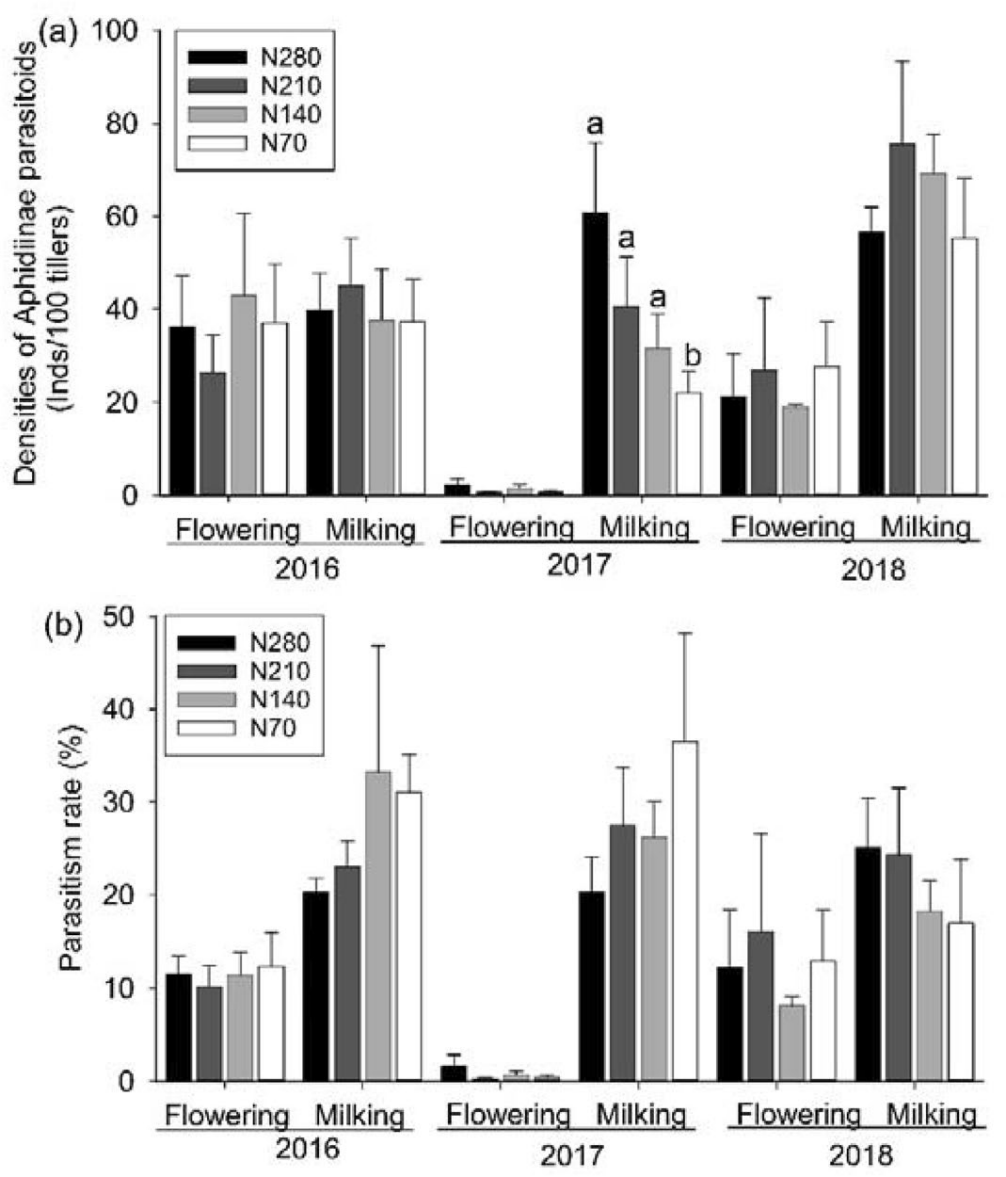
Densities of Aphidiinae parasitoids (**a**) and parasitism rate (**b**) (means + SE) in nitrogen fertilizer plots at the wheat flowering and milking phases during 2016–2018.

The densities of parasitoids in the N70 plots were significantly lower than those in the N280 plots at the milking phase in 2017 (*F*_3, 22_ = 6.02, *P* = 0.004, Fig. 2a). However, the differences between density of parasitoids in the N280 plots and those in the N210 or N140 plots were nonsignificant statistically. The nitrogen levels did not obviously influence the density of parasitoids in either 2016 or 2018.

Nitrogen fertilizer did not significantly influence the parasitism rate in any investigated year (Fig. 2b).

### Effect of decreasing nitrogen fertilizer on the body sizes of parasitoids

**Figure 3:**
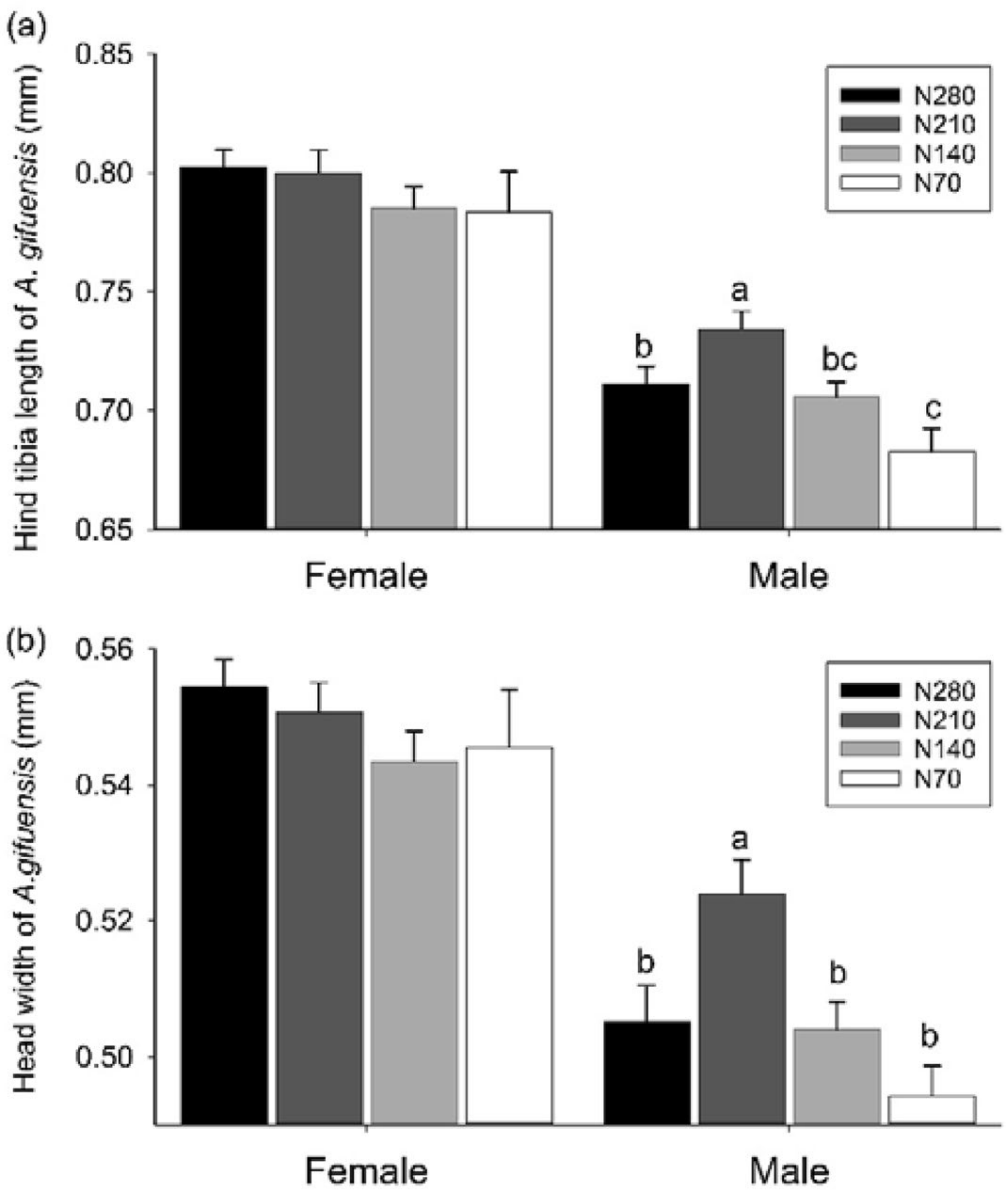
Body size of *A. gifuensis* (**a**: hind tibia length; **b**: head width) (means + SE) in nitrogen fertilizer plots at the wheat flowering phase in 2017.

For the parasitoid *A*. *gifuensis*, the hind tibias of the adult males in the N210 plots were significantly longer than those in the N70, N140, and N280 plots (*F*_3, 121_ = 5.813, *P* = 0.001, Fig. 3a). The head widths of adult males in the N210 plots were significantly greater than those in the N70, N140, and N280 plots (*F*_3, 121_ = 5.481, *P* = 0.001, Fig. 3b). However, nitrogen fertilizer application did not significantly affect the hind tibia lengths or head widths of *A*. *gifuensis* adult females*.*

**Figure 4:**
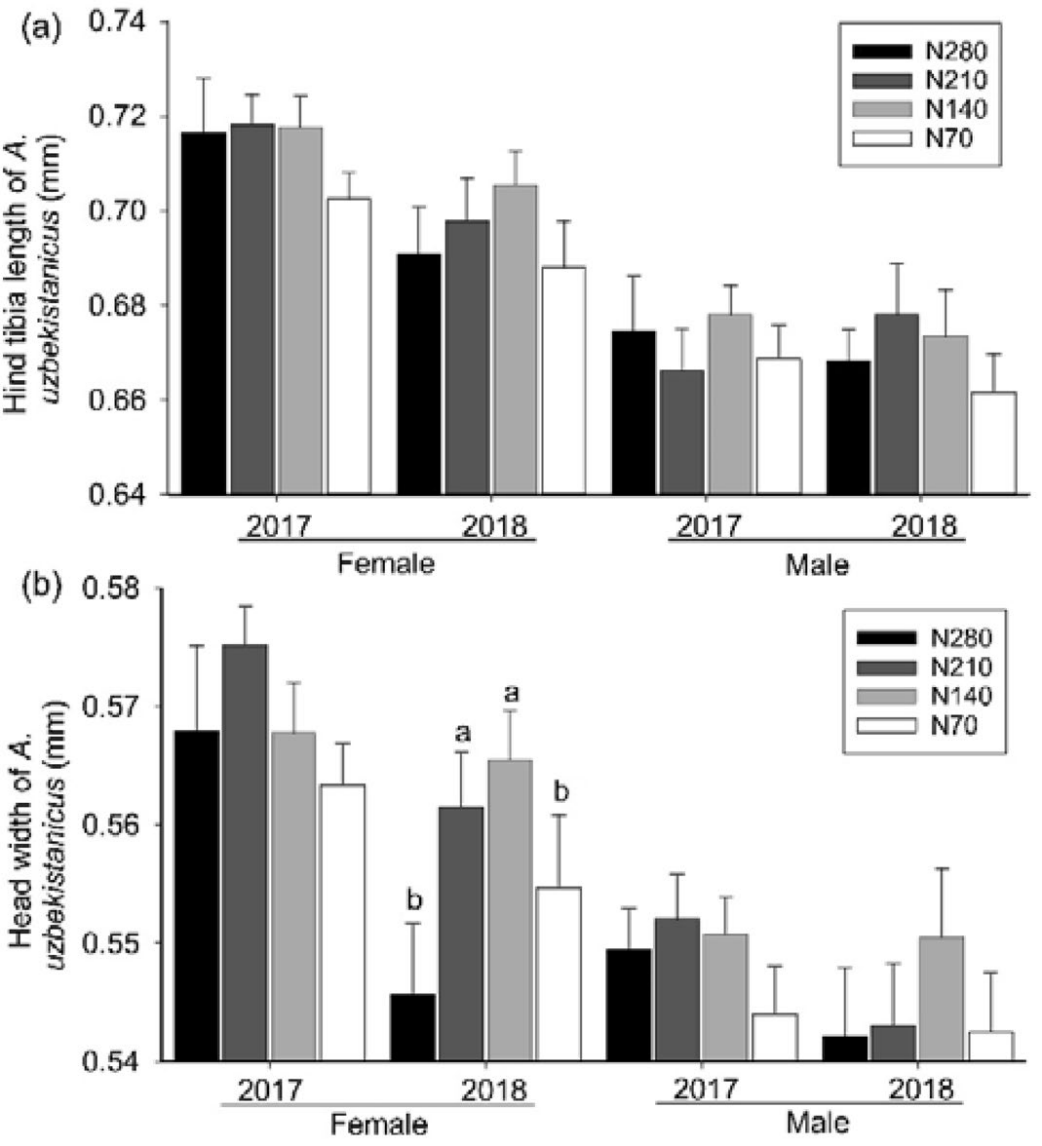
Body size of *A. uzbekistanicus* (**a**: hind tibia length; **b**: head width) (means + SE) in nitrogen fertilizer plots at the wheat flowering phase during 2017–2018.

For the parasitoid *A*. *uzbekistanicus*, the head widths of the adult females in the N140 and N210 plots were greater than those in the N70 and N280 plots in 2018 (*F*_3, 176_ = 3.089, *P* = 0.029, Fig. 4b). However, nitrogen fertilizer application did not significantly affect the hind tibia length or the head width of adult males across all investigated years (Fig. 4a)*.*

### Effect of parasitism rate on the population growth of cereal aphids

**Figure 5:**
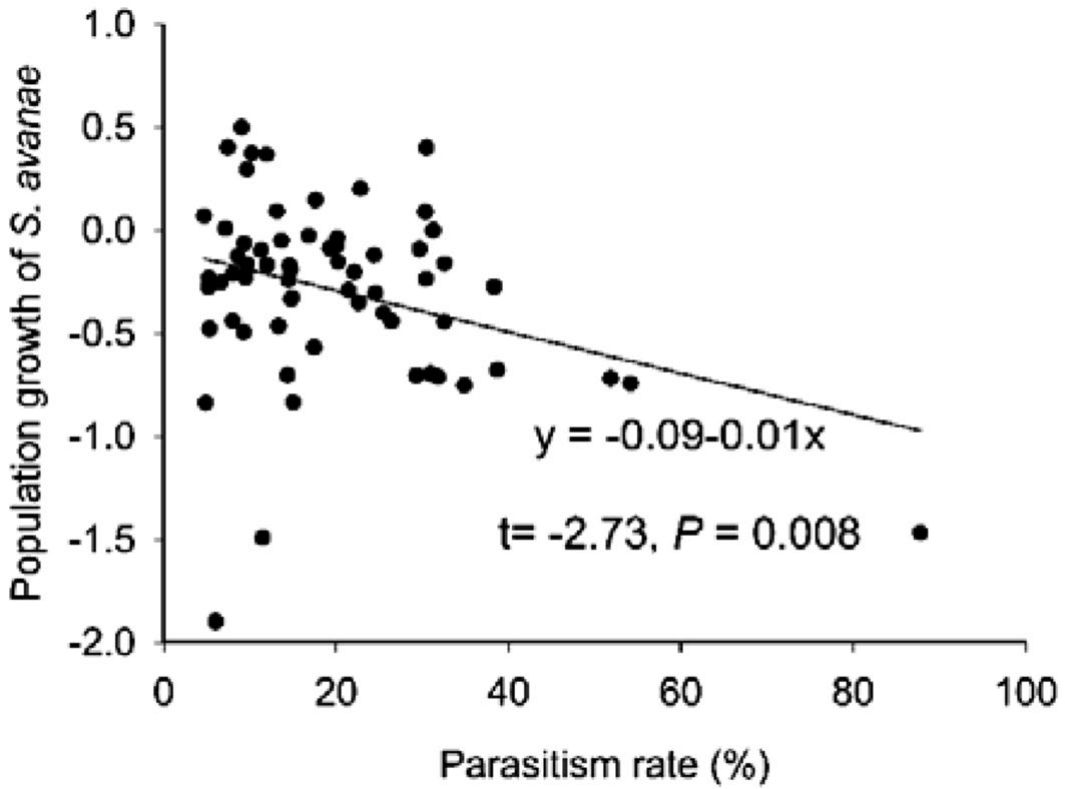
Relationship of population growth of *S. avenae* to parasitism rate in the milking phase during 2016–2018. The population growth of *S. avenae* was calculated as the log_10_(x + 1) transformed density of *S. avenae* at the milking phase minus the log_10_(x + 1) transformed density of *S. avenae* at the flowering phase.

For the cereal aphid *S. avenae,* the densities were affected significantly by wheat growth phase in 2016 (*P* = 0.001) and 2017 (*P* = 0.001), while the effect was not significant in 2018. From flowering to milking phase, the overall densities of *S. avenae* declined by 46% in 2016, declined by 48% in 2017 and increased by 52% in 2018. The interaction between nitrogen levels and wheat phases did not affect significantly the densities of *S. avenae* in the three years (Table 1). The population growth of *S. avenae* was negatively associated with parasitism rate (*df* = 63, *t* = − 2.73, *P* = 0.008, Fig. 5).

**Table 1:** Results of split-plot ANOVA for densities of cereal aphids (individuals per 100 tillers), with wheat phase (P) and nitrogen input (N). Significant influence (P < 0.05) is indicated by the boldface.

Year	Source	df	*S. avenae*		*R. padi*	
			F	Sig	F	Sig
2016	P	1	**12.20**	**0.001**	2.10	0.154
	N	3	0.72	0.544	**3.06**	**0.038**
	P × N	3	0.72	0.547	1.10	0.358
2017	P	1	**14.12**	**0.001**	**11.71**	**0.001**
	N	3	2.59	0.065	**6.71**	**0.001**
	P × N	3	0.528	0.666	0.53	0.663
2018	P	1	3.95	0.062	**21.74**	** < 0.001**
	N	3	1.14	0.361	0.14	0.937
	P × N	3	0.270	0.846	0.17	0.913

For the other cereal aphid *R. padi,* the densities were affected significantly by nitrogen application in 2016 (*P* = 0.038) and 2017 (*P* = 0.001), while the effect was not significant in 2018. From flowering to milking phase, the overall densities of *R. padi* increased by 175% in 2016, 240% in 2017 (*P* = 0.001) and 395% in 2018 (*P* < 0.001). The interaction between nitrogen levels and wheat phases did not affect significantly the densities of *R. padi* in the three years (Table 1).

## Discussion

Through a three-year investigation, we found that a moderate decrease of nitrogen from 280 to 140–210 kg N ha^−1^ did not markedly influence the populations of cereal aphids or the parasitism rate. However, a moderate decrease of nitrogen input from 280 to 140–210 kg N ha^−1^ maximized the fitness of two predominant Aphidiinae parasitoid species, suggesting parasitoid control of cereal aphid would get benefit from the moderate decrease of nitrogen fertilizer. Those results showed that moderately decreasing nitrogen fertilizer could boost the parasitoid control of cereal aphids. Our research suggests that moderately decreasing nitrogen input is qualitatively beneficial to parasitoids but would not control cereal aphids quantitatively.

### Effect of decreasing nitrogen fertilizer on the cereal aphid population

This study demonstrated that nitrogen fertilizer has the potential to positively influence densities of *S. avenae* and *R. padi* among all manipulated nitrogen fertilizer levels (70–280 kg N ha^−1^) (Fig. 1). Similar conclusions have been documented in research linked with aphids, including cereal aphids^5,17,24^. First, the plant usually responds monotonously and positively to nitrogen fertilizer. The percentage of nitrogen in the dry weight of tobacco leaves was positively associated with fertilizer levels^25^. Nitrogen fertilizer in the range of 0–225 kg N ha^−1^ improved nitrogen concentration of canola throughout the growing season^26^. It has been reported that fertilization has a positive influence on plants, indicating a cascading effect on herbivorous pests^24,26,27^. Nitrogen input could enhance the nutritional quality of the host, as nitrogen input increases sugars and amino acids availability for aphids, thereby accelerating the population growth of the herbivores^28,29^. Second, fertilization negatively affects plant defensive responses to herbivores and lessens the amounts of toxins in host plants^27^. For example, nitrogen fertilizer employed for walnut seedlings decreased the allocation to defensive toxins such as juglone, thereby lowering resistance to walnut aphids^30^. Third, fertilization alters the microclimate of crops and thereby contributes to the population growth of aphids^17,31^.

However, only the lowest nitrogen level manipulated in our experiment (70 kg N ha^−1^) significantly reduced the population of cereal aphids compared with the conventional nitrogen level (280 kg N ha^−1^) in 2016 and 2017 (Fig. 1). Those results showed that the magnitude of decreasing fertilizer input from the conventional level (280 kg N ha^−1^) to a moderate level (140–210 kg N ha^−1^) was insufficient to contain the population of cereal aphids. The performance of cereal aphids could remain unaffected when fertilizer input was decreased to a low level, as aphids could adapt to the pressure of deficient nutrition by sucking more strongly^10^. Therefore, to reduce the population of cereal aphids, the nitrogen level should be decreased to 70 kg·N·ha^−1^ or lower. Similarly, as fertilizer was applied to tobacco in the range of 0–200 ppm N, the nymph weights of whiteflies on tobacco plants did not diminish markedly until the nitrogen concentration level was reduced from 200 to 0 ppm N^25^.

Nevertheless, cereal yield responds to nitrogen levels as a negatively accelerating curve based on previous studies^7,9^. Far lower nitrogen input sharply reduces grain yield, and moderate nitrogen fertilizer is always imperative in agricultural production^2,7^. Therefore, the tradeoff between maintaining the essential grain yield and reduction of the pest population would not have been optimized solely by decreasing nitrogen input.

The wheat variety adopted in our experiment was susceptible to cereal aphids. The landscape around our field employed in this experiment was predominated by winter wheat, and thus the landscape was extremely simplified. By comparison, use of a resistant variety and intercropping wheat with another crop mediated the impact of nitrogen input on densities of cereal aphids^10,12^. If these factors are taken into consideration, it then seems more unlikely that the pest population can be controlled solely by decreasing nitrogen input in complex realistic agricultural environments.

### Effect of decreasing nitrogen fertilizer on the densities of parasitoids and parasitism rate

The results showed that the parasitism rate remained unchanged with nitrogen input (Fig. 2), similar to the results of Garratt, who pointed out that fertilizer levels did not affect the parasitism rate in a cereal-aphid-parasitoid system, as the densities of aphids and their parasitoids increased synchronously with the amount of fertilizer^18^. Similar findings were observed in a walnut aphid-Aphidiinae parasitoid system^24^. Mixed results were reported in previous studies^5,11^. The densities of cereal aphids and parasitoids increased when input of nitrogen fertilizer increased from 115 to 170 kg N ha^−1^, while the parasitism rate increased steadily^5^.

Parasitoids are subject to pressures derived from higher trophic level. Coincidental intraguild predation is ubiquitous in the form of parasitized aphids suffering from predation. The effect of coincidental intraguild predation on biocontrol and the abundance of parasitoids remains controversial^32,33^. Importantly, the Aphidiinae parasitoids have the potential to identify the odors of ladybird beetles and reduce searching efficiency by themselves and their offspring, a trait-mediated indirect effect unrelated with the densities of ladybird beetles^34^. It is possible that the behavior of Aphidiinae parasitoids and the parasitism rate could have been mediated indirectly by ladybird beetles and other predators. Furthermore, the hyperparasitoids also could have relieved biocontrol by Aphidiinae parasitoids^35^. Hence, the higher trophic level could relieve the effects of nitrogen levels on densities of parasitoids and the parasitism rate.

### Effect of decreasing nitrogen fertilizer on the body size of Aphidiinae parasitoids

This research has shown that nitrogen fertilizer application impacted the body sizes of the two Aphidiinae parasitoids (Figs. 3, 4). It has been reported that the body sizes of parasitoids increased monotonically with nitrogen fertilizer under low densities of aphids in the laboratory^18,22^, meanwhile the dispersion capacity of parasitoid adults, the fecundity of adult females, the emergence rate, the adult longevity of parasitoids, and the parasitism rate increased with the body sizes of parasitoids^19,20,22^. In contrast to previous reports, this field study found that a moderate decrease in nitrogen application from 280 to 140–210 kg N ha^−1^ maximized the body sizes of parasitoids. The body sizes of parasitoids depend negatively on the abundance of parasitoids and positively on the hosts diversity^19,36,37^. Hence, combining the positive effect of the abundance of aphids and of the nitrogen input with the negative effect of parasitoid abundance, it is assumed that an equilibrium should emerge balancing the positive effect of abundance of aphids and the negative effect of abundance of parasitoids. Analogously, It has been reported that an optimized nitrogen level maximized the ratio of predators to prey in a canola-mustard aphid-predatory gall midge system^26^.

Manipulating nitrogen fertilizer to maximize the fitness of parasitoids plays a crucial role in natural pest control. Increasing the body sizes of parasitoids means greater fertility and dispersal ability of adults^20,21^, higher fitness of offspring^38^, and the resulting greater capacity to control the aphid. Thus, decreasing nitrogen fertilizer from the conventional level to more environmentally-friendly magnitudes (140–210 kg N ha^−1^) could increase the fitness of Aphidiinae parasitoids and boost the biocontrol by parasitoids. Regrettably, this research study did not validate such a viewpoint since the parasitism rate was not maximized under the moderate nitrogen levels. First, there may be hysteresis effects. The parasitoids that were measured for body sizes came from mummies that were sampled in the flowering phases. These parasitoids came into play and mummified cereal aphids after more than ten days. The mummies remained scarce before the flowering phase. Thus, a notable lag occurred and the effect of parasitoid fitness on the parasitism rate could have been unobservable in this study. Second, apart from affecting parasitoid fitness, nitrogen application affected pest fitness. A moderate amount nitrogen maximized the performance of the green peach aphid and the Bertha armyworm^23,39^. A positive relationship between aphid weight and hind tibia length of parasitoids has been reported^18^. Combined with the finding in this study that the body sizes of parasitoids were maximized by moderate nitrogen levels, these results imply that the fitness of cereal aphids also benefited from moderate nitrogen levels. However, the densities of cereal aphids in moderate nitrogen levels were similar to those under higher nitrogen levels, suggesting that there could be a compensation between the effect of nitrogen input on fitness of cereal aphids and the effect of nitrogen input on fitness of parasitoids. Currently, long-term agricultural intensification limited biocontrol of parasitoids^5^. Previous study has reported that the parasitoids were more strongly influenced by agricultural intensification compared to cereal aphids^5,13,14^. If serious agricultural intensification had mediated, for example decreasing nitrogen fertilizer to an optimized extent, the equilibrium between the impact of moderate decreasing nitrogen fertilizer on parasitoids and the counterpart on cereal aphids would be reshaped. Thus, the positive influence of decreasing nitrogen fertilizer on parasitoids would prevail. Coincidentally, such a magnitude of decreasing nitrogen application would maintain the current wheat yield and lessen the potential environmental risks^9^.

### Relationship between the parasitism rate and the population growth of cereal aphids

From flowering to milking phase, the population of the cereal aphid *R. padi* that escaped from Aphidiinae parasitoids increased substantially in both 2017 and 2018, while the population of the cereal aphid *S. avenae* decreased markedly in both 2016 and 2017 (Table 1). Combining the differences between dynamics of the two cereal aphid species with the fact that the Aphidiinae parasitoids rarely parasitize *R. padi* in China^40^, it is apparent that the Aphidiinae parasitoids play a pivotal role in suppressing the cereal aphid *S. avenae*. Furthermore, a higher parasitism rate had a greater suppression effect on the population of the cereal aphid *S. avenae*, in line with previous research^6,14,41^.

### Year-to-year fluctuation of the cereal aphids-Aphidiinae parasitoids interaction

Obvious fluctuations in the cereal aphids-Aphidiinae parasitoids interaction across years have been documented in this study. Such population fluctuations of aphids and their natural enemies are ubiquitous^14,17,42^. It has been assumed that a disadvantageous climate accounted for the fluctuations^17^. The climate changes could not have been manipulated in our study, but they play essential roles in population fluctuations^43^. Climate warming induced an outbreak of the cereal aphids, but the parasitism rate remained unchanged^43,44^. Lack of Aphidiinae parasitoids caused higher populations of the cereal aphid *S. avenae* in a simulated warmed wheat field. However, abundant Aphidiinae parasitoids retained effective suppression of the cereal aphids even when the wheat field was warmed^45^. The synchronization of parasitoids with pests is vitally important for maintaining biocontrol^46^, while climate change has the potential to mismatch the pests with parasitoids and cause strong population fluctuations of pests and natural enemies^47^.

In this study, the parasitism rate was evaluated according to the densities of discernible mummies, a conventional method widely adopted^5,6,24^. We keep in mind that this method neglects the fact that the symptomless aphids that have been parasitized. Consequently, the parasitism rare was underestimated and the annual fluctuations of abundance of the parasitoids and the parasitism rate were magnified, especially early in the season. Molecular detection, which has the capacity to evaluate whether symptomless aphids have been parasitized and if so by which parasitoid species, presents an exceedingly promising alternative for exploring the aphid-parasitoid interaction^11,33^. This burgeoning method should be employed to more accurately evaluate the aphids-parasitoids interaction.

## Conclusion

Agricultural intensification is posing detrimental risks to grain production. Thus, diminishing intensification in farmland via decreasing nitrogen fertilizer input is all-important. This study explored the effects of decreasing nitrogen fertilizer on both cereal aphids and their parasitoids. This study showed that moderately decreasing nitrogen fertilizer, from the conventional nitrogen level (280 kg N ha^−1^) to a moderate level (140–210 kg N ha^−1^), did not significantly influence the densities of two cereal aphids or the parasitism rate. This seemingly implied that the prospects are dim for aphid management by cultural control. However, the parasitoids benefited from a moderate decrease of nitrogen input as the fitness of parasitoids was maximized by a moderate nitrogen level. We hope that a moderate decrease of nitrogen input can be employed to promote parasitoids’ suppression of pests in the near future.

Our experimental field was set within the region where agricultural intensification has become increasingly serious. Agricultural intensification jeopardizes biocontrol and sustainable development of agriculture^5,13^. If this disappointing situation is to be reversed, then the parasitoids should have a greater role in terms of biocontrol. It is expected that an optimal nitrogen treatment could reduce the populations of cereal aphids by improving the abundance and fitness of parasitoids.

## Materials and methods

### The study area

The experiment was conducted at the Yucheng Comprehensive Experiment Station affiliated to the Chinese Academy of Science (36° 57′ N, 116° 36′ E, Altitude 20 m), in northern China. Further details concerning the characteristics of the field station are given in a previous study^8^. A randomized block design with four nitrogen fertilizer levels (70, 140, 210, and 280 kg N ha^−1^, denoted as N70, N140, N210, N280, respectively) was established in 2005. The highest nitrogen level (N280) approaches the characteristic application by local farmers. The middle nitrogen levels (N140 and N210) approach the preliminary criterion that has been recommended (120–170 kg N ha^−1^)^9^. The lowest nitrogen level (N70) is exceedingly low so that the cereal yield sharply declines.

Six repetitions for three nitrogen levels (N70, N140, and N210) and eight repetitions for the N280 level were set. All plots were concentrated in one field surrounded farmer-managed wheat fields. The field was far from natural habitat as well from as wildflowers and weeds. Plots, 10 m × 5 m in size, were isolated with cement walls to prevent unexpected fertilizer turnover between the individual plots.

A susceptible wheat cultivar, Jimai-22, was sown in mid-October in line with traditional local practices. Nitrogen fertilizer was in the form of urea. Half of the dose was applied in the course of tillage prior to sowing, and the other half was applied at the jointing phase.

### Insect investigation

The insect investigation was conducted across 2016–2018. Investigation was performed at two characteristic wheat growth phases, the wheat flowering phase (late April to early May) and the wheat milking phase (early to middle May). The two wheat phases were chosen based on the fact that they represented peak periods of cereal aphids^5^. Five investigating sampling points per plot were chosen based on a five-spot-sampling method. The cereal aphids were identified based on morphological characteristics^48^. The numbers of the two cereal aphid species and mummies were counted from 20 wheat tillers per sampling point. Thus, 100 tillers were surveyed in each plot. All plots were investigated in 2016 and 2017. Half plots, namely four repetitions for the N280 level and three plots for other levels, were investigated in 2018.

### Measurements of body sizes of parasitoids

To investigate the influence of nitrogen fertilizer on the body sizes of Aphidiinae parasitoids during the wheat flowering phase in 2017 and 2018, four plots for the N280 level and three plots for every other level were chosen. After insect investigation at the flowering phase as mentioned above, about 50 mummies were sampled carefully throughout the plot and packaged individually into 0.5-ml centrifuge tube.

The mummies were kept at room temperature in the laboratory until the parasitoids emerged. The newly-emerged Aphidiinae parasitoids were identified to species and sex^40^. Afterwards, the hind tibia length and head width were measured by means of a binocular stereoscope with a scale.

### Statistical analysis

As the parasitoids rarely parasitize *R. padi* in China according to previous report^40^, the parasitism rate was calculated as the densities of mummies (individuals/100 wheat tillers) divided by the summation of the densities of *S. avenae* (individuals/100 wheat tillers) and the densities of mummies:

 

$$P = \frac{{D}_{m}}{{D}_{m}+{D}_{S}}$$

where *P* denotes the parasitism rate; *D*_*m*_ denotes density of mummies, and *D*_*S*_ denotes the density of *S. avenae.*

For analyzing the effect of nitrogen fertilizer in every representative investigated year, the densities of the cereal aphids and parasitoids were transformed by log_10_(x + 1), and the parasitism rates were transformed to arcsine square roots. We utilized one-way ANOVA with an LSD test to compare the means across nitrogen fertilizer levels. The hind tibia lengths and head widths of adults of two parasitoid species, both females and males, were compared based on a similar method. As the *A*. *gifuensis* adults emerging from collected mummies were scarce in 2018, only the data for *A*. *gifuensis* in 2017 were analyzed. The statistical analyses were performed using the SPSS statistical package (version 20.0).

The effects of wheat phase and nitrogen input on the densities of cereal aphids were analyzed using split-plot ANOVA with nitrogen input and wheat phase as the main effect factors (SPSS), with the densities of cereal aphids transformed by log_10_(x + 1) in advance.

Following a previous report^14^, we calculated population growth of *S. avenae* in terms of the log_10_(x + 1)-transformed densities of *S. avenae* at the milking phase minus those at the flowering phase. To evaluate the influence of the parasitism rate on population growth of *S. avenae* during the two wheat phases, linear regression with pooled data for all nitrogen levels across three years was performed using SigmaPlot 14.0, which also was used for drawing the graphs.

## References

[1] Liu N, Li X, Waddington SR (2014). Soil and fertilizer constraints to wheat and rice production and their alleviation in six intensive cereal-based farming systems of the Indian sub-continent and China. Food Secur..

[2] Hawkesford MJ (2014). Reducing the reliance on nitrogen fertilizer for wheat production. J. Cereal. Sci..

[3] Liu X (2011). Nitrogen deposition and its ecological impact in China: An overview. Environ. Pollut..

[4] Clark CM, Tilman D (2008). Loss of plant species after chronic low-level nitrogen deposition to prairie grasslands. Nature.

[5] Zhao ZH, Hui C, He DH, Li BL (2015). Effects of agricultural intensification on ability of natural enemies to control aphids. Sci. Rep..

[6] Thies C, Roschewitz I, Tscharntke T (2005). The landscape context of cereal aphid-parasitoid interactions. Philos. Trans. R. Soc. Lond. B. Biol. Sci..

[7] Hartmann TE (2015). Yield and N use efficiency of a maize-wheat cropping system as affected by different fertilizer management strategies in a farmer's field of the North China Plain. Field Crops Res..

[8] Zheng CY (2019). Effect of coupled reduced irrigation and nitrogen fertilizer on soil mite community composition in a wheat field. Ecol. Evol..

[9] Ma G (2019). Determining the optimal N input to improve grain yield and quality in winter wheat with reduced apparent N loss in the north China plain. Front. Plant Sci..

[10] Fluegel SM, Johnson JB (2001). The effect of soil nitrogen levels and wheat resistance on the Russian wheat aphid, *Diuraphis noxia* (Homoptera: Aphididae). J. Kansas. Entomol. Soc..

[11] Vollhardt IMG (2019). Influence of plant fertilisation on cereal aphid-primary parasitoid-secondary parasitoid networks in simple and complex landscapes. Agric. Ecosyst. Environ..

[12] Wang LY, Cui HY, Chang XY, Zhu MM, Zhao ZH (2020). Increased nitrogen fertilization inhibits the biocontrol activity promoted by the intercropping partner plant. Insect Sci..

[13] Jonsson M (2012). Agricultural intensification drives landscape-context effects on host-parasitoid interactions in agroecosystems. J. Appl. Ecol..

[14] Plećaš M (2014). Landscape composition and configuration influence cereal aphid–parasitoid–hyperparasitoid interactions and biological control differentially across years. Agric. Ecosyst. Environ..

[15] Yang F (2017). Species composition and seasonal dynamics of aphid parasitoids and hyperparasitoids in wheat fields in northern China. Sci. Rep..

[16] Stav G, Blaustein L, Margalit Y (2005). Individual and interactive effects of predator and controphic species on mosquito populations. Ecol. Appl..

[17] Duffield S, Bryson R, Young J, Sylvester-Bradley R, Scott R (1997). The influence of nitrogen fertiliser on the population development of the cereal aphids *Sitobion avenae* (F.) and *Metopolophium dirhodum* (Wlk.) on field grown winter wheat. Ann. Appl. Biol..

[18] Garratt MPD, Leather SR, Wright DJ (2010). Tritrophic effects of organic and conventional fertilisers on a cereal-aphid-parasitoid system. Entomol. Exp. Appl..

[19] Bukovinszky T, van Veen FJ, Jongema Y, Dicke M (2008). Direct and indirect effects of resource quality on food web structure. Science.

[20] Ameri M, Rasekh A, Michaud JP, Allahyari H (2013). Morphometric indicators for quality assessment in the aphid parasitoid, *Lysiphlebus fabarum* (Braconidae: Aphidiinae). Eur. J. Entomol..

[21] Henri DC, van Veen FJF (2011). Body size, life history and the structure of host–parasitoid petworks. Adv. Ecol. Res..

[22] Aqueel MA (2015). Tritrophic interactions between parasitoids and cereal aphids are mediated by nitrogen fertilizer. Insect Sci..

[23] Chesnais Q, Couty A, Catterou M, Ameline A (2016). Cascading effects of N input on tritrophic (plant-aphid-parasitoid) interactions. Ecol. Evol..

[24] Mace KC, Mills NJ (2016). Nitrogen-mediated interaction: A walnut-aphid-parasitoid system. Environ. Entomol..

[25] Pekas A, Wäckers F (2020). Bottom-up effects on tri-trophic interactions: Plant fertilization enhances the fitness of a primary parasitoid mediated by its herbivore host. J. Econ. Entomol..

[26] Fallahpour F, Ghorbani R, Nassiri-Mahallati M, Hosseini M (2019). Plant fertilization helps plants to compensate for aphid damage, positively affects predator efficiency and improves canola yield. J. Pest Sci..

[27] Chen Y, Olson DM, Ruberson JR (2010). Effects of nitrogen fertilization on tritrophic interactions. Arthropod-Plant Interact..

[28] Aqueel M, Leather S (2011). Effect of nitrogen fertilizer on the growth and survival of *Rhopalosiphum padi *(L.) and *Sitobion avenae* (F.) (Homoptera: Aphididae) on different wheat cultivars. Crop Prot..

[29] Gao J, Guo HJ, Sun YC, Ge F (2018). Differential accumulation of leucine and methionine in red and green pea aphids leads to different fecundity in response to nitrogen fertilization. Pest Manag. Sci..

[30] Strugstad M, Despotovski S (2012). A summary of extraction, properties, and potential uses of juglone: A literature review. J. Ecosys. Manag..

[31] Throop H, Lerdau M (2004). Effects of nitrogen deposition on insect herbivory: Implications for community and ecosystem processes. Ecosystems.

[32] Borer ET (2002). Intraguild predation in larval parasitoids: Implications for coexistence. J. Anim. Ecol..

[33] Traugott M, Bell JR, Raso L, Sint D, Symondson WO (2012). Generalist predators disrupt parasitoid aphid control by direct and coincidental intraguild predation. Bull. Entomol. Res..

[34] Frago E, Godfray HC (2014). Avoidance of intraguild predation leads to a long-term positive trait-mediated indirect effect in an insect community. Oecologia.

[35] Gómez-Marco F (2015). Untangling the aphid-parasitoid food web in citrus: Can hyperparasitoids disrupt biological control?. Biol. Control..

[36] Cohen J, Jonsson T, Carpenter S (2003). Ecological community description using the food web, species abundance, and body size. Proc. Natl. Acad. Sci..

[37] Otto S, Rall B, Brose U (2007). Allometric degree distributions facilitate food-web stability. Nature.

[38] Jervis M, Ellers J, Harvey J (2008). Resource acquisition, allocation, and utilization in parasitoid reproductive strategies. Ann. Rev. Entomol..

[39] Weeraddana CS, Evenden ML (2018). Canola nutrition and variety affect oviposition and offspring performance in the generalist herbivore, *Mamestra configurata* (Lepidoptera: Noctuidae). J. Econ. Entomol..

[40] Chen JH, Shi QX (2001). Systematic studies on Aphiidae of China (Hymenoptera: Aphidiinae).

[41] Gagic V (2011). Food web structure and biocontrol in a four-trophic level system across a landscape complexity gradient. Proc. Biol. Sci..

[42] Leather S, Walters K, Dixon A (1989). Factors determining the pest status of the bird cherry-oat aphid, Rhopalo-siphum padi (L.) (Hemiptera: Aphididae), in Europe: A study and review. Bull. Entomol. Res..

[43] Tougeron K, Brodeur J, Le Lann C, van Baaren J (2019). How climate change affects the seasonal ecology of insect parasitoids. Ecol. Entomol..

[44] Derocles SAP (2018). Climate warming alters the structure of farmland tritrophic ecological networks and reduces crop yield. Mol. Ecol..

[45] Dong Z, Hou R, Ouyang Z, Zhang R (2013). Tritrophic interaction influenced by warming and tillage: A field study on winter wheat, aphids and parasitoids. Agric. Ecosyst. Environ..

[46] Gómez-Marco F, Tena A, Jaques JA, García AU (2015). Early arrival of predators controls *Aphis spiraecola* colonies in citrus clementines. J. Pest Sci..

[47] Damien M, Tougeron K (2019). Prey-predator phenological mismatch under climate change. Curr. Opin. Insect Sci..

[48] Blackman RL, Eastop VF (1984). Aphids on The World's Crops: An Identification Guide.

